# A comparative evaluation of two clinical decision support systems for safer prescribing in older adults: an exploratory single-patient case study

**DOI:** 10.3389/fphar.2026.1830248

**Published:** 2026-05-13

**Authors:** Veera Bobrova, Shane Desselle, Jyrki Heinämäki, Daisy Volmer

**Affiliations:** 1 Faculty of Medicine, Institute of Pharmacy, University of Tartu, Tartu, Estonia; 2 Touro University California College of Pharmacy, Vallejo, CA, United States

**Keywords:** aged, clinical decision support systems, community pharmacy, EU(7)-PIM, EURO-FORTA, medication review, polypharmacy, potentially inappropriate medication list

## Abstract

**Introduction:**

The geriatric population face a heightened risk of adverse drug events due to multimorbidity and polypharmacy, yet clinical decision support systems used in community pharmacies often lack explicit geriatric prescribing criteria.

**Methods:**

This exploratory, proof-of-concept single-patient study evaluated the real-world performance of a newly developed Integrated Clinical Decision Support Tool (PIM Tool), which incorporates the EU (7)-PIM and EURO-FORTA frameworks, and compared it with e-Pharmacist, the clinical decision support module currently used in Estonia's national pharmacy system. A single patient case from the EuroAgeism dataset was analysed, with medications assessed independently in both systems.

**Results:**

The PIM Tool identified potentially inappropriate medications based on age, diagnosis, treatment duration, and cumulative risk, whereas e-Pharmacist primarily detected drug-drug interactions, organ-specific cautions, and dose adjustment needs. Several clinically relevant risks, including age-inappropriate long-term therapies and cumulative sedative burden, were primarily identified by the PIM Tool, while interaction-related and monitoring-related risks were captured exclusively by e-Pharmacist. Patient-reported symptoms appeared to align with overlapping risk patterns identified when outputs from both systems were considered together.

**Discussion:**

These findings suggest that clinical decision support systems based on geriatric prescribing criteria and those focused on interaction screening may capture different but complementary medication-related risks. Using both approaches together may support a more comprehensive identification of medication-related problems and deprescribing opportunities in community-dwelling older adults.

## 1 Introduction

Ensuring safe and effective medication use in older adults is a growing priority in European health systems due to population ageing, multimorbidity, and polypharmacy (Kurczewska-Michalak et al., 2021; Davies et al., 2020). Community-dwelling older adults are particularly vulnerable to medication-related problems, as care is often fragmented across multiple prescribers and largely mediated through outpatient encounters and community pharmacies (Kallio et al., 2018; Morin et al., 2018). In this setting, community pharmacists are well positioned to identify medication-related risks and to support medication optimisation through structured medication reviews (MR) and clinical decision support systems (CDSS) (Gillespie et al., 2009; Gillespie et al., 2013).

However, CDSS embedded in routine pharmacy workflows are primarily optimised to detect drug-drug interactions, organ-specific cautions, and dose adjustment needs, yet do not systematically address geriatric prescribing appropriateness, including age-, diagnosis-, and duration-dependent risks (Moon et al., 2023; Wright et al., 2009). Geriatric prescribing frameworks such as the EU (7)-PIM and EURO-FORTA frameworks were developed to address these dimensions and have been shown to support identification of potentially inappropriate medications (PIMs) and deprescribing opportunities in older adults (Renom-Guiteras et al., 2015; Kruger et al., 2021). Despite this, their integration into routine community pharmacy practice remains limited and heterogeneous across different European settings, with varying degrees of implementation of tools such as STOPP/START, PRISCUS, as well as other similar tools used in different healthcare systems (Anlay et al., 2024).

Estonia provides a relevant real-world context for exploring these challenges, given its advanced national digital health infrastructure and long-standing implementation of electronic health and pharmacy information systems (World Health Organization, 2024). Community pharmacists operate within a nationwide digital pharmacy infrastructure (NOOM) that includes the e-Pharmacist support module, which provides interaction-based alerts, organ-specific precautions, and monitoring guidance (World Health Organization, 2024; Astro Baltics Ltd., 2024). In parallel, pharmacist-led MR services have been introduced since 2019, strengthening the clinical role of pharmacists in primary care (Tuula et al., 2024). These developments highlight the need for CDSS that extend beyond interaction screening and incorporate geriatric-specific risk assessments.

To address this unmet need, we developed an Integrated Clinical Decision Support Tool (PIM Tool) that combines the EU (7)-PIM and EURO-FORTA frameworks into a unified, pharmacy-oriented structure (Bobrova et al., 2022). The PIM Tool classifies medications according to geriatric risk level, accounting for age appropriateness, diagnosis relevance, treatment duration, and cumulative risk burden. Unlike e-Pharmacist, which focuses on drug interactions and organ-specific safety signals, the PIM Tool provides a complementary perspective based on geriatric prescribing criteria.

Despite the growing use of CDSS in community pharmacy, there is limited understanding of how different types of geriatric CDSS complement each other in identifying medication-related risks in older adults. To date, there is limited evidence on how effectively routine pharmacy systems identify geriatric prescribing risks, or how their outputs compare with those of a dedicated geriatric tool in real-world community pharmacy practice. The Estonian context therefore serves as a real-world setting in which this broader question can be explored.

This exploratory single-patient study aimed to explore the outputs of the newly developed PIM Tool in identifying potentially inappropriate medications in an older adult with multimorbidity and polypharmacy, and to compare its outputs with those generated by the e-Pharmacist module. We hypothesised that the two systems would generate complementary findings: PIM Tool would identify age-, diagnosis-, and duration-dependent appropriateness risks, while e-Pharmacist would highlight interaction-related and monitoring-related safety concerns. Rather than testing effectiveness, this study analytically compares the types of medication-related risks identified by two fundamentally different CDSS paradigms when applied to the same real-world patient. As a single-patient exploratory analysis, the purpose is not generalisation but to inform future research and guide the potential integration of geriatric prescribing frameworks into routine community pharmacy practice.

## 2 Materials and methods

### 2.1 Study design

This study used an exploratory, proof-of-concept comparative case design in a community pharmacy context. The approach prioritised feasibility, interpretability, and differences in risk identification between CDSS under real-world complexity (polypharmacy, multimorbidity, and patient-reported symptoms), rather than estimation of effectiveness or prevalence. Accordingly, findings are hypothesis-generating and not generalisable.

### 2.2 Data source and case selection

The case was sampled from Estonia’s contribution to the EuroAgeism Horizon 2020 dataset of community-dwelling adults aged ≥65 years (EuroAgeism Consortium, 2020). The dataset served solely as a sampling frame. The EuroAgeism dataset included 310 participants, of whom 164 had polypharmacy (≥5 medications), and 107 had documented at least one PIM. The case analysed in this study was selected from this subgroup. A purposive sampling approach was used. The aim was to identify an information-rich case that reflects a typical older adult seen in community pharmacy practice. The selected patient had a relatively high number of medications and multiple PIMs, along with common chronic conditions and medications routinely encountered in outpatient care. Cases with rare, highly specialised, or acute conditions were not considered. The patient was functionally independent and representative of someone regularly attending a community pharmacy.

Extracted variables included a complete medication list (prescription and over-the-counter medicines, and supplements), patient-reported diagnoses, symptoms during the preceding 3 months, and self-reported adherence. No additional clinical data (e.g., laboratory values) were available, reflecting the realities of community pharmacy practice in Estonia.

### 2.3 Clinical background and patient overview

The case involved an older community-dwelling adult with multimorbidity and polypharmacy, receiving multiple medications across therapeutic domains (Table 1). The patient was independent in daily activities and was receiving routine outpatient care. Partial medication adherence was reported, mainly due to forgetfulness, along with occasional short-term memory difficulties without a diagnosed cognitive impairment. Over the preceding 3 months, the patient experienced symptoms including orthostatic dizziness, chest discomfort, shortness of breath, gastrointestinal disturbances, dehydration, and chronic musculoskeletal pain. The combination of older age, polypharmacy, partial adherence, and multi-domain symptom burden constituted a clinically relevant baseline for subsequent medication risk assessment using CDSS.

**TABLE 1:** General characteristics and medication profile of the patient.

Section	Variable/Medication	Description
1. Patient characteristics	Age range	70–75 years
	Clinical profile	Community-dwelling older adult with multimorbidity and polypharmacy
	Cognitive status	Occasional memory difficulties reported
	Medication adherence	Partial adherence reported
	Main symptoms (past 3 months)	Orthostatic dizziness, chest discomfort, peripheral edema, shortness of breath, gastrointestinal disturbance, dehydration, chronic musculoskeletal pain
	Functional status	Independent in daily activities
2. Medication profile grouped by therapeutic class	Antidiabetic agents	
	Metformin	Diabetes (E11); long-term use; standart dosing
	Liraglutide	Diabetes (E11); long-term use; standart dosing
	Alpha-lipoic acid (supplement)	Polyneuropathy (G63); long-term use; oral administration
	Cardiovascular drugs	
	Bisoprolol	Hypertension (I10); long-term use; standart dosing
	Telmisartan	Hypertension (I10); long-term use; standart dosing
	Nifedipine (short-acting)	Hypertension (I10); long-term use; PRN
	Amiodarone	Arrhythmia (I48); unknown duration; standart dosing
	Dabigatran	Atrial fibrillation (I48); long-term use; standart duration
	Warfarin	Atrial fibrillation (I48); long-term use; INR-guided dosing
	Rosuvastatin	Dyslipidaemia (E78); unknown duration; standart dosing
	Pain management and musculoskeletal agents	
	Paracetamol + codeine	Soft-tissue disorder (M79); long-term use; PRN; fixed-dose combination
	Tramadol + dexketoprofen	Soft-tissue disorder (M79); long-term use; fixed-dose combination
	Oxycodone	Gonarthrosis (M17); long-term use; low-dose regimen
	Tizanidine	Gonarthrosis (M17); long-term use; standart dosing
	Neurologic and psychiatric agents	
	Mirtazapine	Sleep disorder (G47); long-term use; night-time dosing
	Duloxetine	Polyneuropathy (G63); long-term use; standart dosing
	Gastrointestinal protection	
	Pantoprazole	GERD (K21); long-term use; standart dosing
	Esomeprazole	GERD (K21); long-term use; standart dosing
	Anti-infective	
	Amoxicillin + clavulanic acid	Dental infection (K04); short-term use; standart dosing
	Respiratory agent	
	Codeine + guaifenesin	Chronic cough (R05); long-term use; standart dosing

Abbreviations: ICD-10, International Classification of Diseases; PRN, as needed; INR, international normalized ratio; GERD, gastroesophageal reflux disease.

### 2.4 Medication verification

Reported medications were verified using NOOM (World Health Organization, 2024; Astro Baltics Ltd., 2024), Estonia’s national pharmacy information system, which provides access to electronic prescriptions, dispensing history, product characteristics, and integrated CDSS alerts. As community pharmacists in Estonia do not have access to laboratory or diagnostic results, assessments relied on patient-reported information, NOOM records, and system outputs. Where no alerts were generated in the multi-drug view, individual Summaries of Product Characteristics were reviewed to confirm the absence of system-identified safety warnings.

### 2.5 Clinical decision-support systems

The PIM Tool combines the EU (7)-PIM and EURO-FORTA frameworks into a unified geriatric appropriateness schema. Medications were classified as high-, moderate-, or low-risk PIMs, or as non-PIMs, based on age appropriateness, diagnosis relevance, treatment duration, cumulative central nervous system burden, and potential for harm.

The integration of the EU (7)-PIM and EURO-FORTA frameworks within the PIM Tool follows a structured approach. Each medication is first assessed independently according to both frameworks. EURO-FORTA classifications provide the primary indication-based categorisation (A-D), while EU (7)-PIM criteria are used to identify medications with established age-related inappropriateness. Final risk levels (high, moderate, low) are assigned through a synthesis of these steps, considering the highest level of concern, as well as contextual factors such as treatment duration and cumulative pharmacodynamic burden. In practice, this follows a rule-based approach in which concordant high-risk classifications across frameworks result in high-risk PIM designation, while partial or discordant classifications are assigned to moderate or low-risk categories. A detailed description of the tool development and classification logic is available in our previous work (Bobrova et al., 2022).

The classification reflects a precautionary and screening-oriented approach that is consistent with community pharmacy practice, where access to detailed clinical information is often limited. In this setting, medications may still be flagged as PIMs based on established geriatric prescribing frameworks, even when certain patient-specific parameters, such as renal function, are not available. Rather than providing a definitive judgement for an individual patient, this type of classification is intended to highlight possible areas of concern and to support further clinical evaluation when needed. The tool does not generate drug–drug interaction alerts.

To support interpretation of these classifications in practice, the assigned risk levels are intended to reflect the relative clinical priority of each medication. High-risk PIMs point to medications with a clearly unfavorable benefit-risk balance in older adults and typically require prompt reassessment or deprescribing. Moderate-risk PIMs indicate medications that may be appropriate in selected cases but require careful review of indication, dose, and patient-specific factors. Low-risk PIMs highlight potential concerns mainly related to prolonged use of cumulative effects and are more likely to require monitoring or periodic review rather than immediate action.

The e-Pharmacist module, integrated within NOOM, was used to identify drug-drug interactions, organ-specific cautions (renal, hepatic, cardiovascular), dose-adjustment recommendations, duplicate therapy, and monitoring needs. Internally, the module draws on structured knowledge bases (e.g., INXBASE and RISKBASE), with outputs accessed exclusively *via* the e-Pharmacist interface.

Unlike the PIM Tool, the e-Pharmacist module does not assign medications to predefined geriatric risk categories. Instead, it generates individual alerts related to interactions, organ-specific risks, and monitoring needs, leaving clinical prioritisation to the pharmacist. At the same time, its alert-based structure reflects a focus on identifying acute and mechanism-based risks that may require immediate attention in clinical practice.

### 2.6 Analytical procedure

**FIGURE 1:**
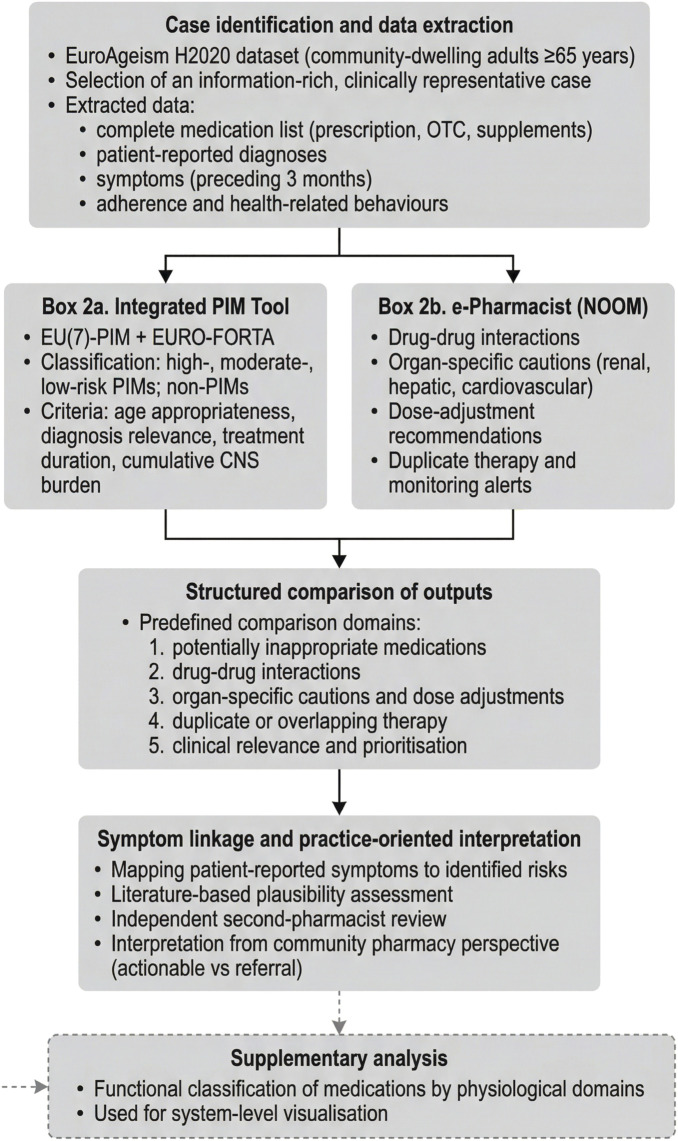
Analytical workflow of the exploratory, proof-of-concept comparative case study.

A predefined four-stage workflow was followed (Figure 1). First, the complete medication regimen was assessed independently using both systems. Where relevant, medication dosing and treatment duration were available in the original dataset and were considered during the assessment. However, detailed dosing information is not presented in Table 1 to reduce the risk of potential patient re-identification. The assessment was based on established geriatric prescribing criteria applied to the available clinical context. Second, outputs were compared across five predefined domains: (i) identification of potentially inappropriate medications, (ii) detection of drug-drug interactions, (iii) identification of organ-specific cautions and dose-adjustment needs, (iv) duplicate or overlapping therapy, and (v) clinical relevance and prioritisation of flagged risks. The final assessment represents an integrated interpretation of outputs from both CDSS (PIM Tool and e-Pharmacist) and should not be interpreted as equivalent to PIM classification only. Medications defined as non-PIM by the PIM Tool may still be categorized as carrying some level of risk in the final assessment when additional safety considerations, such as interaction burden or monitoring requirements identified by the e-Pharmacist system, are present. Third, patient-reported symptoms were mapped to plausible medication-related risks using literature-based adverse-effect profiles and interaction plausibility. The symptom-medication mapping and overall interpretation were performed by the lead author based on the available clinical information and outputs from both CDSS. NOOM-based outputs were obtained using the e-Pharmacist module and used for subsequent analysis. No formal inter-rater reliability assessment was conducted, as the study was designed as an exploratory, single-case analysis.

Symptom-medication associations should be interpreted as clinically plausible hypotheses to guide pharmacist intervention, not as evidence of causal attribution. Non-pharmacological contributors to reported symptoms could not be excluded. Finally, findings were interpreted from a community pharmacy perspective, focusing on which risks could realistically be identified and addressed during routine pharmacy encounters, and which would require referral to a prescriber.

In addition, an author-led functional classification of all medications across major physiological domains (cardiovascular, endocrine, bleeding-related, gastrointestinal) was performed to support the system-level visualisation presented in Table 2.

**TABLE 2:** Functional classification of medication effects across physiological systems.

Medication	Anticoagulation/Bleeding	Cardiovascular and renal	Central nervous system	Gastrointestinal
High-risk PIMs				
Nifedipine (short-acting)	**-**	**☑**	**-**	**-**
Codeine + guaifenesin	**-**	**-**	**☑**	**o**
Tramadol + dexketoprofen	**☑**	**o**	**☑**	**☑**
Moderate-risk PIMs				
Amiodarone	**-**	**☑**	**-**	**-**
Bisoprolol	**-**	**☑**	**-**	**-**
Dabigatran	**☑**	**o**	**-**	**-**
Mirtazapine	**-**	**-**	**☑**	**-**
Duloxetine	**-**	**-**	**☑**	**o**
Tizanidine	**-**	**o**	**☑**	**-**
Oxycodone	**-**	**-**	**☑**	**o**
Low-risk PIMs				
Pantoprazole	**-**	**-**	**-**	**☑**
Esomeprazole	**-**	**-**	**-**	**☑**
Not PIMs/Neutral medications (may contribute in combinations)				
Metformin	**-**	**-**	**-**	**o**
Liraglutide	**-**	**-**	**-**	**o**
Telmisartan	**-**	**o**	**-**	**-**
Rosuvastatin	**-**	**o**	**-**	**o**
Paracetamol + codeine	**-**	**-**	**☑**	**o**
Amoxicillin + clavulanic acid	**-**	**-**	**-**	**o**
Alpha-lipoic acid	**-**	**-**	**-**	**-**
Warfarin	**☑**	**-**	**-**	**-**

Abbreviations: PIM, potentially inappropriate medication.

Each medication was mapped according to reported effects on major physiological domains, including cardiovascular, endocrine, bleeding-related, and gastrointestinal systems. Symbols indicate the presence (**☑**), absence (**−**), or unclear or inconsistently reported evidence (**o**) of system-specific effects based on summaries of product characteristics and published clinical sources.

### 2.7 Ethics

The EuroAgeism dataset received national ethical approval (298/T-2). This secondary analysis used pseudonymised data only, and no additional approval was required under local regulations. To minimize the risk of potential participant re-identification, patient characteristics and medication details presented in the Table 1 were reported in de-identified and aggregated form.

## 3 Results

### 3.1 PIM tool outputs

Using the integrated EU (7)-PIM and EURO-FORTA frameworks, 11 of the patient’s 20 medications (55%) were classified as potentially inappropriate (Table 3). Three medications were identified as high-risk PIMs: short-acting nifedipine, chronic use of codeine as an antitussive, and long-term treatment with a fixed-dose combination of tramadol and dexketoprofen. Six medications were classified as moderate-risk PIMs (amiodarone, bisoprolol, dabigatran, duloxetine, mirtazapine, and tizanidine), while pantoprazole and esomeprazole were considered low-risk PIMs, primarily due to treatment duration.

**TABLE 3:** Comparative medication assessment using PIM Tool and e-Pharmacist outputs.

Medication	PIM tool classification (EU (7)-PIM + EURO-FORTA)	PIM risk level	e-Pharmacist findings (NOOM)	Final assessment (combined interpretation on both CDSS)
Nifedipine (short-acting, PRN)	Short-acting form = high-risk; class D (avoid)	High-risk PIM	Risk of severe hypotension and cardiac events	Avoid short-acting form; consider long-acting substitute
Codeine + guaifenesin	Codeine as cough suppressant = high-risk; long-term use unsafe	High-risk PIM	Sedation, constipation, dependence, respiratory risk	High-risk; recommend discontinuation/substitution
Tramadol + dexketoprofen	NSAID (high-risk) + opioid (moderate-risk); chronic use ↑ risk	High-risk PIM	GI, renal, and CNS adverse effects; caution with anticoagulants	High-risk combination; avoid chronic use
Amiodarone	Listed for cumulative toxicity; class C (use with caution)	Moderate-risk PIM	Warned about thyroid, hepatic, QT, and drug–drug interactions	Moderate risk; requires close monitoring
Bisoprolol	β-blockers may cause bradycardia/CNS effects; B–C class (appropriate with review)	Moderate-risk PIM	Heart rate and BP monitoring advised; interaction with amiodarone/nifedipine	Moderate risk; dose slightly high; ECG checks needed
Dabigatran	Limited elderly data; bleeding risk; renal dependence; class C	Moderate-risk PIM	Bleeding and renal monitoring recommended	Moderate risk; acceptable with close monitoring
Mirtazapine	Beneficial alternative but class C (caution for sedation/falls)	Moderate-risk PIM	Sedation, weight gain, and cumulative CNS load noted	Moderate risk; appropriate if monitored
Duloxetine	SNRIs → fall, bleed, seizure risk; class C	Moderate-risk PIM	Advised BP and sodium monitoring; watch for interactions	Moderate risk; suitable for neuropathic pain with caution
Tizanidine	Centrally acting relaxant; hypotension and sedation risk; not in FORTA.	Moderate-risk PIM	Reported drowsiness and hepatic risk; dose monitoring needed	Moderate risk; reassess long-term use
Pantoprazole	Low-risk; review if > 8 weeks; class B	Low-risk PIM	Long-term PPI use linked to infection/nutrient risk	Low risk; review duration and need
Esomeprazole	Same as pantoprazole; long-term caution	Low-risk PIM	Suggests periodic review; similar safety profile	Low risk; suitable with ongoing evaluation
Warfarin	Safe with INR control; class B (appropriate with monitoring)	Not PIM	Many interactions; INR stability essential	Low risk; appropriate under INR supervision
Metformin	Beneficial medication; safe unless severe renal or heart failure; class B (beneficial)	Not PIM	Minor interaction with bisoprolol → mild risk of hypoglycaemia; advise glucose monitoring	Appropriate; continue with glucose checks
Liraglutide	Beneficial (class B); appropriate for T2DM; no PIM listing	Not PIM	Emphasized renal monitoring; no new safety issues	Suitable for continued use with renal checks
Alpha-lipoic acid (supplement)	Not listed; insufficient geriatric data; neutral status	Not PIM	No alerts or contraindications	Neutral; monitor necessity and tolerance
Amoxicillin + clavulanic acid	Not included in PIM lists; short course appropriate	Not PIM	No geriatric-specific risk; routine GI monitoring only	Safe short-term antibiotic use
Telmisartan	Not flagged as PIM; appropriate indication and dose	Not PIM	No major warnings; monitor BP and renal function	Safe and appropriate antihypertensive
Rosuvastatin	No PIM warning; class B (beneficial)	Not PIM	Routine muscle/liver monitoring suggested	Safe for long-term use with monitoring
Paracetamol + codeine	Codeine analgesic not PIM unless >2 weeks continuous; D only for COPD.	Not PIM (context caution)	Mild sedation and constipation; risk ↑ with CNS drugs	Appropriate analgesic; moderate caution if long-term
Oxycodone	Lower delirium risk vs. other opioids; class B (beneficial)	Not PIM	Routine constipation/fall precautions advised	Safe at low dose with standard monitoring

Abbreviations: PIM, potentially inappropriate medication; EU (7)-PIM, European Union 7 potentially inappropriate medications list; EURO-FORTA, European Fit fOR, the aged classification; NOOM, pharmacy management software system (Astra Baltics); NSAID, non-steroidal anti-inflammatory drug; PPI, proton pump inhibitor; INR, international normalized ratio; QT, QT, interval; GI, gastrointestinal; SOB, shortness of breath; CNS, central nervous system; BP, blood pressure; ECG, electrocardiogram; CDSS, clinical decision support system.

The remaining nine medications (45%) were classified as non-PIMs within the PIM Tool assessment.

Although warfarin was not identified as a PIM, it was retained as a low-risk medication in the final assessment due to its interaction profile and monitoring requirements identified through the combined outputs of both CDSS (Table 3).

### 3.2 e-pharmacist outputs

The NOOM e-Pharmacist module generated safety alerts for 14 of the 20 medications (70%), while six agents did not produce alerts in the multi-drug review (Table 4). The alerts clustered into four main domains of medication-related risk.

**TABLE 4:** NOOM-generated safety and interaction findings for 14 medicines included in the analysis.

Risk profile	Involved active substances	Main NOOM alerts/Interactions	Clinical relevance for an older patient (≥65 years)	Example of NOOM recommendation
1. Anticoagulation and bleeding risk	Warfarin, dabigatran, amiodarone, paracetamol + codeine, tramadol + dexketoprofen, amoxicillin + clavulanic acid, rosuvastatin	Multiple interactions increasing INR and bleeding risk; amiodarone increases dabigatran bioavailability; warfarin affected by several drugs including paracetamol and antibiotics	Major cumulative bleeding risk from concurrent anticoagulant and interacting drugs; most critical signal for this patient	Avoid concurrent use or monitor INR and bleeding closely
2. CNS depression and serotonergic effects	Oxycodone, codeine + guaifenesin, paracetamol + codeine, tramadol + dexketoprofen, duloxetine	Sedation, psychomotor impairment, constipation; risk of serotonin syndrome with duloxetine + opioids	High fall risk, confusion, and impaired coordination; directly relevant to reported dizziness	Avoid alcohol and driving; monitor for sedation and serotonin toxicity
3. Cardiovascular and renal risk	Telmisartan, bisoprolol, amiodarone, nifedipine, rosuvastatin, metformin	Additive bradycardia and hypotension (amiodarone + bisoprolol); NSAID–RAAS interaction (telmisartan + dexketoprofen); possible renal burden	Increased hemodynamic instability and renal impairment risk due to combined antihypertensive therapy	Monitor BP, HR, renal function; avoid dehydration and unnecessary NSAID use
4. Adherence- and therapy course-related risks	Amiodarone, duloxetine, oxycodone, paracetamol + codeine, amoxicillin + clavulanic acid	Risks from abrupt discontinuation, accumulation (amiodarone), or incomplete courses (antibiotics)	Relevant for patient with partial adherence and mild cognitive issues	Do not discontinue without medical consultation; ensure follow-up
5. Age- and population-specific cautions	Warfarin, amiodarone, oxycodone, duloxetine, tramadol + dexketoprofen, bisoprolol, nifedipine	“Use with caution ≥65 years,” sedation, bradycardia, hypotension; avoid alcohol or driving	Reflects NOOM’s partial overlap with PIM criteria, highlighting typical geriatric risks	Use lowest effective dose, monitor for orthostasis and sedation
6. No specific NOOM alerts generated (SPC accessible separately)	Liraglutide, tizanidine, mirtazapine, pantoprazole, esomeprazole, alpha-lipoic acid	Not flagged during multi-drug analysis; only basic SPC information available		

Abbreviations: NOOM, pharmacy management software system (Astra Baltics); INR, international normalized ratio; CNS, central nervous system; NSAID, non-steroidal anti-inflammatory drug; SPC, summary of product characteristics; PIM, potentially inappropriate medication.

Anticoagulation and bleeding risk constituted the most prominent cluster, with repeated alerts related to warfarin- and dabigatran-based therapy and interactions with co-medications such as amiodarone and NSAIDs, emphasising cumulative bleeding risk and the need for monitoring.

A second cluster involved central nervous system (CNS) depression and opioid-related risks, including sedation, constipation, impaired psychomotor performance, and driving restrictions, as well as interaction-related warnings for opioid–serotonergic combinations such as tramadol and duloxetine.

Cardiovascular and renal safety concerns formed the third cluster, with alerts highlighting additive bradycardia and hypotension from combined cardiovascular therapies and increased renal burden associated with concomitant ARB and NSAID use.

Finally, therapy duration- and adherence-related cautions were generated across several medications, including warnings against abrupt discontinuation and risks of accumulation during long-term use.

### 3.3 Comparative analysis of PIM tool and e-pharmacist outputs

The two CDSS produced complementary but conceptually distinct outputs. The PIM Tool identified age-, diagnosis-, and duration-dependent prescribing risks, including high-risk PIMs such as short-acting nifedipine and chronic codeine use, which were not consistently prioritised by the NOOM system unless additional interacting agents were present.

In contrast, the NOOM e-Pharmacist module focused on interaction-based, organ-specific, and monitoring-oriented safety alerts, particularly in relation to anticoagulation, cardiovascular stability, and renal function. This distinction was also reflected in the final assessment, which represented the interpretation of the outputs from both systems. Accordingly, some medications classified as non-PIM by the PIM Tool, such as warfarin, were still retained as low-risk medications in the final assessment (Table 3).

A summary comparison of outputs is presented in Table 5, and clustering of risks across physiological domains is illustrated in Table 2.

**TABLE 5:** Complementary strengths of the Integrated PIM Tool and NOOM in medication risk assessment.

Aspect	Integrated PIM tool (EU (7)-PIM + EURO-FORTA)	NOOM pharmacy support system	Contribution to this patient’s assessment
Identifying PIMs (high, moderate, low risk)	Yes, provides explicit classification for older adults	No, NOOM does not identify PIMs	Only the integrated PIM tool identified potentially inappropriate medications
Age-specific appropriateness (≥65 years)	Yes, entire system built around geriatric criteria	No, NOOM provides general adult precautions	Integrated PIM tool identified age-sensitive risks (short-acting nifedipine, codeine for cough)
Diagnosis-based appropriateness	Yes, *via* EURO-FORTA (indication matters)	No, diagnoses not used in appropriateness assessment	Integrated PIM tool marked diagnosis-linked concerns (e.g., NSAIDs in chronic conditions)
Duration-sensitive risks (short vs. long term)	Yes, distinguishes long-term appropriateness	No, NOOM does not classify by duration	Integrated PIM tool flagged chronic PPI and chronic NSAID use as potentially inappropriate
Drug-class level evaluation (opioids, NSAIDs, sedatives)	Yes, classification includes drug families	Limited, NOOM focuses on single drugs	Integrated PIM tool identified cumulative sedative burden
Identification of safe/Not PIM medications	Yes, confirms appropriateness	No, NOOM does not designate a drug as “appropriate”	Helps differentiate “problems” vs. “stable therapies”
Interaction checking	Limited	Yes, detailed drug-drug interactions	NOOM detected metabolic and pharmacodynamic interactions not included in the PIM tool
Dose adjustment guidance	Not included	Yes, provides renal dose adjustments, hepatic contraindications	NOOM highlighted dose-dependent safety considerations (dabigatran, metformin)
Monitoring recommendations	Limited	Yes, suggests lab tests and monitoring frequency (e.g., renal function, INR)	NOOM added practical follow-up steps for anticoagulants and renally-cleared drugs
Symptom-based alerts	Not included	Yes, links patient symptoms to medication effects	Helped match constipation, dizziness, SOB with opioids and cardiovascular drugs
Detailed pharmacological safety notes	Not designed for this	Extensive	NOOM added nuances (QT risk, hepatic monitoring, GI warnings)
Context-dependent risk evaluation	Yes, appropriateness depends on age, diagnosis, duration	No, considers pharmacology but not appropriateness	Both systems together provided a fuller picture
Overall strength	Geriatric appropriateness and structured PIM categorization	Mechanistic safety, interactions and monitoring guidance	Complementary: Neither system is sufficient alone; together they produce a complete assessment

Abbreviations: PIM, potentially inappropriate medication; EU (7)-PIM, European Union 7 potentially inappropriate medications list; EURO-FORTA, European Fit fOR, the aged classification; NOOM, pharmacy management software system (Astra Baltics); NSAID, non-steroidal anti-inflammatory drug; PPI, proton pump inhibitor; INR, international normalized ratio; QT, QT, interval; GI, gastrointestinal; SOB, shortness of breath; CNS, central nervous system.

### 3.4 Symptom linkage and clinical plausibility

Patient-reported symptoms were mapped to plausible medication-related risk clusters identified by both systems (Table 6). Orthostatic dizziness and chest discomfort aligned potentially with the use of short-acting nifedipine and the combined effects of amiodarone and bisoprolol, which may contribute to hypotension and bradycardia.

**TABLE 6:** PIM classification and potential relationship to the patient’s current symptoms.

Medication	Mechanism of risk	Relevant patient symptoms	Notes on combinations
High-risk PIMs			
Nifedipine (short-acting)	Sudden BP drop; reflex tachycardia	Orthostatic dizziness; chest discomfort	Potentiated by bisoprolol and amiodarone due to heart-rate suppression
Codeine + guaifenesin	Sedation, constipation, respiratory depression	GI disturbance, dizziness, shortness of breath	Sedation may be amplified by mirtazapine, oxycodone, paracetamol + codeine, tizanidine
Tramadol + dexketoprofen	GI bleeding, renal impairment, CNS effects	GI disturbance, dizziness; dehydration	GI risk may be increased with long-term PPIs masking symptoms; bleeding risk may be increased with warfarin/dabigatran
Moderate-risk PIMs			
Amiodarone	Bradycardia, QT prolongation, thyroid dysfunction	Orthostatic dizziness; shortness of breath	Bradycardia risk may be increased with bisoprolol + nifedipine
Bisoprolol	Bradycardia, hypotension	Orthostatic dizziness; peripheral edema	May interact with amiodarone and nifedipine, enhancing hypotension
Dabigatran	Bleeding risk	No overt bleeding reported, dehydration may increase risk	Bleeding risk may be increased with NSAIDs (dexketoprofen)
Mirtazapine	Sedation, orthostatic hypotension	Orthostatic dizziness	Additive with opioids, tizanidine, codeine products
Duloxetine	Hyponatraemia, dizziness	Orthostatic dizziness	Potential interaction with tramadol (serotonin syndrome risk)
Tizanidine	Sedation, hypotension	Orthostatic dizziness	Additive with opioids + codeine products; BP-lowering with antihypertensives
Oxycodone	Sedation, constipation, respiratory depression	GI disturbance, orthostatic dizziness, shortness of breath	Strong synergy with codeine cough syrup and mirtazapine

Medication	Mechanism of risk	Relevant patient symptoms
Low-risk PIMs		
Pantoprazole	Long-term PPI: Infection risk, malabsorption	Dehydration, GI disturbance (possible PPI-related)
Esomeprazole	Same as above	Same as above

Medication	Comments	Combination notes
Not PIMs (but may contribute to symptoms in combinations)		
Metformin	Generally safe; mild GI effects	GI disturbance possible, worsened by PPIs
Liraglutide	GI upset, early satiety	May contribute to weight changes
Telmisartan	Generally well tolerated	Hypotension risk may increase with bisoprolol + nifedipine
Rosuvastatin	Muscle pain	Musculoskeletal pain could worsen if statin-associated myopathy is present
Warfarin	Bleeding if INR unstable	Requires monitoring; interactions may increase bleeding risk
Paracetamol + codeine	Sedation, constipation (if frequent use)	Adds to opioid burden (oxycodone, cough syrup)
Amoxicillin + clavulanic acid	GI symptoms during course	Short-term; limited relevance to current symptom profile
Alpha-lipoic acid	Neutral	No known contributions
Pantoprazole/Esomeprazole	As above	Chronic use + NSAIDs may mask early GI symptoms
Metformin + PPIs	-	Can contribute to B12 deficiency (long-term)
Oxycodone + codeine syrup + mirtazapine + tizanidine	-	May contribute to dizziness, sedation, GI disturbance

Abbreviations: PIM, potentially inappropriate medication; BP, blood pressure; QT, QT, interval; GI, gastrointestinal; PPI, proton pump inhibitor; INR, international normalized ratio; NSAID, non-steroidal anti-inflammatory drug; CNS, central nervous system; B12, vitamin B12.

Dizziness and cognitive symptoms were consistent with cumulative CNS depressant effects from mirtazapine, tizanidine, opioid-containing therapies, and duloxetine, increasing fall risk. Gastrointestinal disturbances and dehydration aligned with opioid-induced bowel dysfunction, NSAID-related effects from dexketoprofen, and long-term proton pump inhibitor use. Chronic musculoskeletal pain despite tramadol/dexketoprofen suggested limited analgesic benefit relative to gastrointestinal and renal risk.

Overall, symptoms reflected overlapping pharmacodynamic effects across cardiovascular, CNS, and gastrointestinal medication clusters rather than a single causative agent, underscoring cumulative medication burden and the value of integrated risk assessment.

## 4 Discussion

### 4.1 Interpretation of findings and relation to existing evidence

This case study illustrates how applying a geriatric-specific prescribing appropriateness tool in a real-world community pharmacy setting may reveal medication-related risks that are not systematically identified by interaction-based CDSS alone. Although the e-Pharmacist module within the NOOM system successfully highlighted clinically important drug-drug interactions, monitoring needs, and organ-specific safety concerns, the PIM Tool provided complementary insights by identifying age-, diagnosis-, and duration-dependent prescribing risks that are particularly relevant for older adults with polypharmacy. Together, these outputs highlighted overlapping risk clusters, particularly in cardiovascular, central nervous system, gastrointestinal, and anticoagulation domains, underscoring the complexity of polypharmacy in older adults.

The identification of 55% of the patient’s medications as potentially inappropriate, including several high- and moderate-risk PIMs, is consistent with previous studies reporting a high prevalence of potentially inappropriate prescribing among community-dwelling older adults (Opondo et al., 2012). Importantly, many of the high-risk PIMs identified in this case, such as short-acting nifedipine, chronic opioid use for non-pain indications, and long-term exposure to non-steroidal anti-inflammatory medicines, are well recognised in geriatric pharmacotherapy guidelines but may remain underprioritised in systems primarily focused on interaction screening (American Geriatrics Society Beers Criteria Update Expert Panel, 2023).

In contrast, e-Pharmacist generated strong alerts for anticoagulation therapy, additive cardiovascular effects, renal burden, and opioid-related monitoring needs. These risks are less explicitly represented within appropriateness frameworks such as EU (7)-PIM and EURO-FORTA, where medications like warfarin are not classified as PIMs but may still require careful monitoring due to their safety profile. The differential emphasis illustrates that the two systems reflect distinct underlying logics: interaction-based CDSS prioritise acute, mechanism-based risks, whereas geriatric appropriateness tools highlight chronic, cumulative, and age-dependent risks.

Mapping patient-reported symptoms to identified risks revealed clinically plausible associations. Orthostatic dizziness and chest discomfort corresponded to short-acting nifedipine and additive effects of amiodarone and bisoprolol. Dizziness and cognitive symptoms aligned with cumulative central nervous system burden from opioids, mirtazapine, and tizanidine. Gastrointestinal disturbances reflected opioid-induced bowel dysfunction, non-steroidal anti-inflammatory drug-related mucosal irritation, and long-term proton pump inhibitor use. These patterns reinforce the concept that adverse outcomes in older adults often arise from cumulative pharmacodynamic effects rather than isolated drug interactions, an insight more readily captured by appropriateness frameworks than by standard drug-drug interaction alerts.

As this analysis is based on a single patient case, these findings should be interpreted as illustrative rather than generalisable.

### 4.2 Symptom-medication plausibility and cumulative risk burden

When the system-level distribution of medication effects is considered alongside the medications identified as high- and moderate-risk PIMs, a clinically plausible relationship emerges between cumulative medication burden and the patient’s reported functional complaints, rather than a single causative medication. Medications contributing to high- and moderate-risk PIM classifications clustered predominantly within central nervous system, cardiovascular, anticoagulation, and gastrointestinal domains, which correspond closely to the patient’s symptoms of orthostatic dizziness, and gastrointestinal disturbances. Appropriateness frameworks were particularly useful in highlighting such cumulative risks, while e-Pharmacist contributed essential insights related to interactions and organ function.

### 4.3 Implications for community pharmacy practice

From a practical perspective, this study highlights the potential value of integrating geriatric-specific appropriateness tools into routine community pharmacy workflows. Pharmacists are often the last healthcare professionals to review a patient’s complete medication profile, particularly for older adults who are not regularly hospitalised and who receive care across multiple prescribers (Jokanovic et al., 2016). In this context, the PIM Tool may support pharmacists in identifying medications that warrant reassessment, deprescribing discussions, or referral to prescribers, even in the absence of overt interaction alerts from e-Pharmacist.

Importantly, several risks identified through geriatric appropriateness assessment in this case were not associated with acute interaction warnings and therefore might remain unnoticed during routine dispensing without a structured medication review. The combined use of e-Pharmacist and a geriatric appropriateness tool may help pharmacists differentiate between risks requiring immediate action, such as interaction- or monitoring-related alerts, and those warranting planned medication review or discussion with prescribers, such as long-term age-inappropriate therapies without acute safety signals.

The findings emphasise that geriatric appropriateness tools should not replace existing interaction-based systems. Instead, the complementary strengths observed in this case suggest that combined use of interaction-focused CDSS, such as e-Pharmacist, and age-specific appropriateness assessment, such as the PIM Tool, provides a more comprehensive evaluation of medication-related risk than either approach alone. This perspective is particularly relevant for medication review services and pharmacist-led interventions aimed at improving medication safety in older adults.

In routine use, the two systems may be applied in a complementary sequence. Interaction-based CDSS, such as e-Pharmacist, are typically used at the point of dispensing to identify immediate safety concerns, including drug-drug interactions, contraindications, and monitoring requirements. The PIM Tool can then support a more structured review of ongoing therapy, drawing attention to age-related appropriateness, treatment duration, and pharmacodynamic burden. This distinction is particularly relevant in the geriatric population, where CDSS designed for the general patient population do not systematically capture age-related prescribing risks.

At the same time, practical implementation challenges should be considered. Introducing additional decision-support layers into routine workflow may increase time burden for pharmacists and contribute to alert fatigue if not carefully prioritised. In busy community pharmacy settings, tools that generate excessive or non-specific alerts risk being overlooked. Future implementation efforts should therefore focus on integrating such tools into existing systems, optimising the process, and ensuring that outputs remain clinically relevant and usable in daily practice.

### 4.4 Conceptual interpretation of decision-support system outputs

A useful way to interpret the findings of this study is through a broader conceptual categorisation of CDSS (Sutton et al., 2020). Medication-related CDSS can be grouped into three major types based on their underlying logic: interaction-focused systems, appropriateness-focused systems, and hybrid or emerging multi-domain systems. The e-Pharmacist module represents the first category, prioritising acute, mechanism-based risks such as drug-drug interactions, organ-specific dose adjustments, and monitoring needs. In contrast, the PIM Tool exemplifies appropriateness-focused CDSS, which identify age-, diagnosis-, and duration-related prescribing concerns and cumulative pharmacodynamic burdens that are highly relevant for older adults but often overlooked by interaction-based systems. Hybrid or emerging CDSS combine both approaches, integrating explicit appropriateness criteria with interaction databases and, increasingly, predictive analytics to capture multidimensional risk profiles. Framing CDSS within this taxonomy highlights that the two tools evaluated in this study reflect complementary decision-support paradigms, helping to explain their different outputs and reinforcing the value of integrating geriatric appropriateness logic into routine pharmacy systems to achieve a more comprehensive assessment of medication-related risk.

### 4.5 Strengths and limitations

The primary strength of this study lies in its real-world, case-based design, which allowed detailed exploration of how different decision-support tools perform when applied to a complex medication regimen typical of community-dwelling older adults. The use of a validated geriatric appropriateness framework and comparison with a nationally implemented pharmacy system further strengthens the relevance of the findings.

However, several limitations must be acknowledged. As a single-case study, the findings are not generalisable and should be interpreted as exploratory and illustrative. Symptom-medication associations were based on patient-reported information and literature-supported plausibility rather than objective clinical or laboratory confirmation. In addition, the PIM Tool does not assess drug-drug interactions, which limits direct comparability with e-Pharmacist outputs and underscores the importance of combined tool use.

### 4.6 Future directions

Future research should extend this approach to larger samples and evaluate whether systematic integration of geriatric appropriateness tools into community pharmacy practice leads to measurable improvements in prescribing quality, symptom burden, and patient-centred outcomes. Further work is also needed to explore digital integration of such tools within existing pharmacy information systems to support scalable, routine use.

## 5 Conclusion

This exploratory proof-of-concept case study suggests that interaction-focused CDSS alone may not capture geriatric-specific prescribing risks in older adults with multimorbidity and polypharmacy. The PIM Tool complemented routine alerts by identifying age-, diagnosis-, and duration-dependent appropriateness concerns aligned with the patient’s symptoms. Although hypothesis-generating, these findings indicate that combining geriatric appropriateness frameworks with existing pharmacy systems may support more comprehensive medication review and risk prioritisation. Larger studies are needed to evaluate the generalisability and clinical impact of these observations.

## Data Availability

The data analyzed in this study were obtained from the EuroAgeism Horizon 2020 project dataset. Access to the dataset is restricted due to ethical and privacy considerations. Requests to access these data should be directed to the corresponding EuroAgeism project coordinators, subject to approval and data-sharing agreements. Requests to access these datasets should be directed to fialovad@faf.cuni.cz.
